# A New Approach for Modeling Mixed Lubricated Piston-Cylinder Pairs of Variable Lengths in Swash-Plate Axial Piston Pumps

**DOI:** 10.3390/ma14195836

**Published:** 2021-10-06

**Authors:** Bo Zhao, Xinqing Hu, Haifeng Li, Yonghui Liu, Baocheng Zhang, Qingbing Dong

**Affiliations:** 1 Department of Mechanical and Electrical Engineering, Ocean University of China, Qingdao 266100, China; hxq@stu.ouc.edu.cn (X.H.); liuyonghui@ouc.edu.cn (Y.L.); zbc2014088@ouc.edu.cn (B.Z.); 2 Key Laboratory of Ocean Engineering of Shandong Province, Ocean University of China, Qingdao 266100, China; 3 Power Plant Operation Department, Naval Submarine Academy, Qingdao 266100, China; ytulihaifeng@163.com; 4 State Key Laboratory of Mechanical Transmission, Chongqing University, Chongqing 400030, China

**Keywords:** transient mixed lubrication, multibody dynamics, variable piston-cylinder length, swash-plate axial piston pump, leakage

## Abstract

The swash-plate axial piston pump is one of the most widely used pumps due to its simplicity and compactness in structure. In such a pump, the piston-cylinder system plays a crucial role, with its lubrication characteristics greatly affecting the overall pumping performance. A new numerical approach is proposed in this study for modeling mixed lubricated piston-cylinder interfaces of variable lengths in swash-plate axial piston pumps in the framework of multibody dynamics. The approach couples the hydrodynamic mixed lubrication model of the piston-cylinder interface with the multibody dynamics model of the piston pump. The lubrication model is established with a transient average Reynolds equation considering asperity contacts and is solved with the finite element method to derive the hydrodynamic forces of the lubricated pair, while the multibody dynamics model is established with Lagrangian formalism by considering hydrodynamic forces as external forces. Results for piston-cylinder interfaces of variable lengths in swash-plate axial piston pumps are presented, and the impacts of cylinder length and the tilt angle of the swash plate on the tribological performances of the interface are discussed. The results indicate that increasing the cylinder length can improve the stability and wear resistance of the piston, but it can exacerbate the frictional power loss. Moreover, although enlarging the tilt angle of the swash plate can effectively increase pump displacement, it can easily lead to serious friction, wear, and leakage problems. Consequently, the tilt angle of the swash plate should be carefully selected in practical applications.

## 1. Introduction

Due to its simple and compact structure, the swash-plate axial piston pump is widely used in hydraulic systems. As one of the fundamental tribo-pairs of piston pumps, the piston-cylinder interface heavily affects the survival and overall performance of the pump, not only because it plays a role in sealing and bearing, but also because it serves as the main source of friction and fluid leakage [1,2]. Moreover, under heavy load conditions, the lateral force from the swash plate can easily induce mixed lubrication between the piston and cylinder, which further increases power consumption and shortens the service life of the pump [3]. Therefore, with piston pumps developing towards high power density and high reliability, it is of great significance to deeply study the piston-cylinder pair for the purpose of optimizing its tribological performance and achieving long-lasting maintenance-free operation.

Considerable and extensive studies have been conducted on the lubrication characteristics of the piston-cylinder tribo-pair in the piston pump. Based on the lubrication conditions described by the Stribeck curve, Manring et al. [4] proposed a model for the friction prediction of the piston-cylinder interface. Ivantysynova et al. [5,6] conducted a series of investigations on the lubrication performances of the piston-cylinder interface, with consideration of heat transfer and thermal elastic deformation phenomena. They also introduced a geometric multigrid algorithm to solve the Reynolds equation for the piston-cylinder interface [7]. Meike et al. [8] obtained the pressure and temperature fields of the piston-cylinder interface with a fluid-structure interaction (FSI) method. However, the above studies mainly involved pumps with fixed lubrication interface lengths between the piston and cylinder. In applications, the interface length was either fixed or varied depending on the cylinder length and pump stroke, and exerted a significant impact on pump performance.

In the last decade, many scholars have also investigated the lubrication performance of the piston-cylinder interface with variable lengths in swash-plate axial piston pumps. Ma et al. [9] introduced an equal-displacement-step method. Through the kinematic analysis of the piston, Jiang et al. [10] proposed an FSI method for modeling the grooved piston-cylinder pair. Sun et al. [2] modeled the piston-cylinder interface with consideration of the adaptive eccentricity and the force balance of the piston. Wang et al. [1,11,12] predicted the lubrication and leakage performances of variable-length piston-cylinder interfaces in a swash-plate axial piston pump, considering piston structure, piston kinematics and dynamic load. Nie et al. [13] proposed an elasto-hydrodynamic lubrication model for the piston-cylinder interface in a seawater hydraulic axial piston pump. However, the above methods for the lubrication study of the variable-length piston-cylinder interface simplified the force state and the eccentric motion of the piston [2]. Since the eccentric motion can directly affect the lubrication performances of the interface, a more comprehensive model is urgently needed.

In recent decades, the multibody dynamics method has attracted more and more attention in modeling mechanical systems with lubricated joints. This method can avoid complicated mechanical model derivation and ensure high calculation accuracy and efficiency [14,15]. Since the swash-plate axial piston pump can be regarded as a typical multibody system, its tribological performance can be predicted in the framework of multibody dynamics. Flores [14] was the pioneer in applying the Reynolds equation to the multibody system. To realize the complete coupling of a lubrication model and dynamics model of a multi-body mechanical system, Zhao et al. [16,17,18,19,20] proposed fully coupling methods to model the internal combustion engine (ICE) with consideration of the lubrication joints. However, compared with the above-mentioned lubricated joints in the ICE, the lubrication inside the piston-cylinder interface in the swash-plate axial piston pump is more complex because its lubrication interface length is variable in most cases, and two primary relative motions between the piston and cylinder liner always exist: translation and rotation. So far, predictions regarding the performance of the piston-cylinder interface in the swash-plate axial piston pump have rarely been reported in the framework of multibody dynamics.

In this paper, to predict accurately the performance of piston-cylinder interfaces of variable lengths in swash-plate axial piston pumps, a novel approach is presented by coupling the lubrication model of the interface with the multibody dynamics model of the pump system. The lubrication model is built with a transient average Reynolds equation that considers asperity contact [21], while the multibody dynamics model is established by Lagrangian formalism. This approach can facilitate the calculation of the eccentric motion of the piston while considering the effect of roughness on lubrication performance in the mixed lubrication regime, and can also simulate lubrication inside the varying piston-cylinder interface, which involves a changing domain and moving boundaries. In this way, investigations are carried out regarding the lubrication and internal leakage performances of a piston-cylinder interface in a swash-plate axial piston pump, as well as their dependence on cylinder length and the tilt angle of the swash plate. The proposed approach provides a theoretical basis for improving lubrication performance, reducing friction and internal leakage while increasing the life cycle of the piston-cylinder interface.

## 2. The Mixed Lubrication Model of a Piston-Cylinder Tribo-Pair

**Figure 1:**
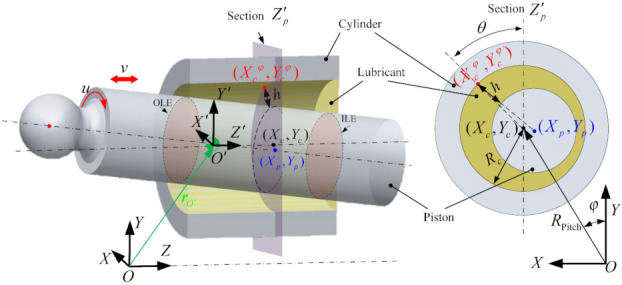
The piston-cylinder misalignment position and oil film thickness definition.

Figure 1 presents a lubricated piston-cylinder interface. The existence of the fluid in the clearance builds up the hydrodynamics forces to separate the piston and cylinder. In this section, a transient lubrication model is introduced to evaluate the hydrodynamics forces.

### 2.1. The Hydrodynamic Mixed Lubrication Model

It is known from the field of fluid mechanics that the load capacity of the oil film is derived from the wedge effect and the squeeze film effect [22,23]. As the relative motion occurs in the lubrication system, the oil film pressure will build up to keep the elements apart. Possible asperity contacts may interrupt the lubrication and break down the lubricant film when the oil film has a very small thickness [24]. In this study, the oil film pressure is evaluated based on the average Reynolds equation proposed by Patir and Cheng [21,25]. In this equation, the lubricant is assumed to be an incompressible Newtonian fluid and the flow is laminar. Moreover, the density and viscosity of the lubricant are assumed to be constant in the direction of the oil film thickness. By introducing the pressure-flow and shear-flow factors into the equation to consider the influence of roughness on the average pressure and average flow of a lubricant between surfaces, the average Reynolds equation can be expressed as

(1) $$\frac{\partial }{\partial x}\left(\phi_{x}\frac{h^{3}}{12\mu }\frac{\partial p}{\partial x}\right)+\frac{\partial }{\partial y}\left(\phi_{y}\frac{h^{3}}{12\mu }\frac{\partial p}{\partial y}\right)=\frac{u}{2}\left[\phi_{c}\frac{\partial h}{\partial x}+\sigma \frac{\partial \phi_{s}}{\partial x}\right]+\frac{v}{2}\left[\phi_{c}\frac{\partial h}{\partial y}+\sigma \frac{\partial \phi_{s}}{\partial y}\right]+\phi_{c}\frac{\partial h}{\partial t}$$

where μ is the dynamic viscosity of oil. h and p are the oil film thickness and presure, respectively. x and y are the local coordinate axes of the unwrapped fluid film thickness of the interface along the circumferential and axial directions of the piston, respectively. They are expressed as $x=\theta R_{p},\;y=Z^{\prime },\;z=h$. Here, $R_{P}$ is the radius of the piston, and θ the angular coordinate. The local coordinate system $O^{\prime }\text{-}X^{\prime }Y^{\prime }Z^{\prime }$ is parallel to the global coordinate system O-XYZ, with its origin located at the center of mass (COM) of the piston, as shown in Figure 1. *t* is the time which is used to considering the squeeze effect of the oil film. v and u denote the axial and circumferential sliding velocity of the piston relative to the cylinder, respectively. σ is the variance of the composite surface roughness, which can be computed from the standard deviation of the roughness height of the 2 sliding surfaces. $\phi_{x}$ and $\phi_{y}$ are the pressure-flow factors along the x and y directions, respectively, reflecting the resistance of the roughness to the lubricant flowing through the rough surface. The shear-flow factor $\phi_{s}$ serves as an additional flow resulting from the small gradient pressure caused by the roughness of 2 relatively sliding surfaces. The contact factor $\phi_{c}$ equates the ratio of the non-contact area with the nominal lubrication area in physical significance [26]. These factors are functions of oil film thickness and are sensitive to the statistical properties of the combined roughness and direction of the surface roughness [21,25]. In this work, the rough surfaces are assumed to take on the Gaussian distribution of roughness heights with the isotropic roughness structure. These factors can be determined according to Patir and Cheng’s works [21,25].

With the assumption that the piston rotates relative to the cylinder with a speed comparable to the shaft speed [2,5] (i.e., the piston will not rotate in the global coordinate system), the circumferential sliding velocity of the piston relative to the cylinder can be expressed as

(2) $$u=\omega R_{P}$$

where ω is the shaft speed of the pump.

The oil film thickness distribution can be determined by the relative position between piston and cylinder, and expressed as

(3) $$h(x,y)=\sqrt{{\left(X_{p}-X_{c}^{\varphi }\right)}^{2}+{\left(Y_{p}-Y_{c}^{\varphi }\right)}^{2}}-R_{P}$$

and

(4) $$\begin{array}{l} X_{p}(z_{p})=X_{O^{\prime }}+y\theta_{y}\\ Y_{p}(z_{p})=Y_{O^{\prime }}-y\theta_{x}\\ X_{c}(\varphi )=R_{\mathrm{Pitch}}\sin\varphi +R_{c}\sin\theta \\ Y_{c}(\varphi )=R_{\mathrm{Pitch}}\cos\varphi +R_{c}\cos\theta \end{array}$$

where $R_{\mathrm{Pitch}}$ is the pitch radius of the cylinder block. $R_{c}$ is the inner radius of the cylinder, and its difference from $R_{P}$ defines the nominal clearance of the piston-cylinder interface c. $\left(X_{O^{\prime }},Y_{O^{\prime }},Z_{O^{\prime }}\right)$ is the position of the COM of the piston in the global coordinate system O-XYZ. $\theta_{x}$ and $\theta_{y}$ are the inclination angle of the piston axis in O-XYZ. φ is the shaft angle of the pump.

According to Equations (3) and (4), the squeeze part of the Reynolds equation is

(5) $$\frac{\partial h(x,y)}{\partial t}=\sin\theta \left(-{\dot{X}}_{O^{\prime }}-y{\dot{\theta }}_{y}+\omega R_{\mathrm{Pitch}}\cos\varphi \right)-\cos\theta \left({\dot{Y}}_{O^{\prime }}\text{-}y{\dot{\theta }}_{x}+\omega R_{\mathrm{Pitch}}\sin\varphi \right)$$

Since the magnitude orders of oil film pressure and thickness in Equation (1) are quite different, the calculation precision can be greatly influenced. Thus, the dimensionless method is adopted to address this problem, which can be written as

(6) $$\theta =\frac{x}{R_{P}},\bar{h}=\frac{h}{c},\bar{y}=\frac{y}{R_{\mathrm{Pitch}}},\bar{\sigma }=\frac{\sigma }{c},\bar{v}=\frac{v}{\omega R_{\mathrm{Pitch}}},\bar{p}=\frac{pc^{2}}{6R_{P}{}^{2}\mu \omega },\bar{\dot{h}}=\frac{2}{c\omega }\frac{\partial h}{\partial t}$$

The dimensionless Reynolds equation can be written as

(7) $$\begin{array}{l} \frac{\partial }{\partial \theta }\left(\phi_{x}{\bar{h}}^{3}\frac{\partial \bar{p}}{\partial \theta }\right)+\frac{R_{P}{}^{2}}{R_{\mathrm{Pitch}}{}^{2}}\frac{\partial }{\partial \bar{y}}\left(\phi_{y}{\bar{h}}^{3}\frac{\partial \bar{p}}{\partial \bar{y}}\right)=\\ \;\;\;\;\;\;\;\;\;\;\;\;\;\;\;\;\;\;\;\;\;\left[\phi_{c}\frac{\partial \bar{h}}{\partial \theta }+\bar{\sigma }\frac{\partial \phi_{s}}{\partial \theta }\right]+\bar{v}\left[\phi_{c}\frac{\partial \bar{h}}{\partial \bar{y}}+\bar{\sigma }\frac{\partial \phi_{s}}{\partial \bar{y}}\right]+\frac{\phi_{c}}{\pi }\bar{\dot{h}} \end{array}$$

In order to solve the Reynolds equation, the Reynolds boundary is applied to the lubrication model of the piston-cylinder interface and can be written as [27]

(8) $$\left\{\begin{array}{c} \left.\begin{array}{c} \begin{array}{l} p(x,y)=0\;\;\;\;\;\;\;\;\;\;\;\;\;\;\;\;\;\;\;\;y=y_{OLE}\\ p(x,y)=P_{s}\;\;\;\;\;\;\;\;\;\;\;\;\;\;\;\;\;\;\;y=y_{ILE} \end{array}\\ p(0,y)=p(2\pi R_{p},y)\;\;\;\;\;\;\;\;\;\;\;\;\;\;\;\; \end{array}\right\}\;\;The\;1st\;boundary\;condition\\ \left.\begin{array}{l} \frac{\partial p}{\partial x}(0,y)=\frac{\partial p}{\partial x}(2\pi R_{p},y)\;\;\\ \frac{\partial p}{\partial y}(x,y)=0\;\;\;\;\;\;\;\;\;y=y_{OLE},y_{ILE}\; \end{array}\right\}\;\;The\;2nd\;boundary\;condition\;\;\;\; \end{array}\right.$$

where $y_{ILE}$ and $y_{OLE}$ correspond to the inner lubrication edge (ILE) and outer lubrication edge (OLE) in the local coordinate system $O^{\prime }\text{-}X^{\prime }Y^{\prime }Z^{\prime }$, respectively. $P_{s}$ is the supply pressure from the displacement chamber. It should be mentioned that this Reynolds boundary neglects oil film reformulation at its boundary. In cases with strong transient effects [28], taking for example the pump with microscopic shape and texture of the piston surface, the cavitation flow can affect the hydrodynamics pressure significantly, and the cavitation boundary with mass-flux continuity at the film reformation boundary is expected to be adopted to further improve the accuracy of the method.

### 2.2. The FEM-Based Model for Solving the Reynolds Equation

In comparison with the finite difference method (FDM), the main advantages of the FEM lie in its strong adaptability, fewer restrictions by geometric shape, arbitrary selection of element size and nodes, and high calculation accuracy [29]. In this study, Equation (7) is solved with FEM, and can be rewritten as

(9) $$\frac{\partial }{\partial \bar{x}}\left(\xi \frac{\partial \bar{p}}{\partial \bar{x}}\right)+\vartheta \frac{\partial }{\partial \bar{y}}\left(\zeta \frac{\partial \bar{p}}{\partial \bar{y}}\right)-\psi =0$$

where

(10) $$\xi =\phi_{x}{\bar{h}}^{3},\;\;\;\zeta =\phi_{y}{\bar{h}}^{3},\;\;\vartheta ={\left(\frac{R_{P}}{R_{\mathrm{Pitch}}}\right)}^{2},\psi =\left[\phi_{c}\frac{\partial \bar{h}}{\partial \theta }+\bar{\sigma }\frac{\partial \phi_{s}}{\partial \theta }\right]+\bar{v}\left[\phi_{c}\frac{\partial \bar{h}}{\partial \bar{y}}+\bar{\sigma }\frac{\partial \phi_{s}}{\partial \bar{y}}\right]+\frac{\phi_{c}}{\pi }\bar{\dot{h}}$$

**Figure 2:**
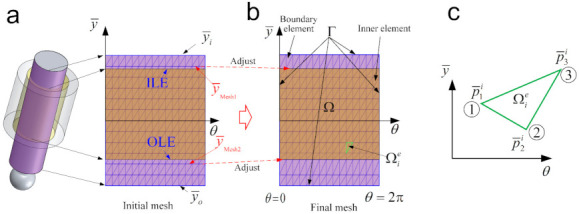
The meshing diagram: (**a**) The initial mesh for the whole domain, (**b**) the final mesh after the fine adjustment, and (**c**) the diagram of an element.

According to the Galerkin theory, the integration over the whole lubrication domain Ω can be built as

(11) $$\iint_{\Omega }Ν\left(\frac{\partial }{\partial \theta }\left(\xi \frac{\partial \bar{p}}{\partial \theta }\right)+\vartheta \frac{\partial }{\partial \bar{y}}\left(\zeta \frac{\partial \bar{p}}{\partial \bar{y}}\right)-\psi \right)d\theta d\bar{y}=0$$

where Ν is the shape function of the discrete elements. With the integration using the Green’s theorem, Equation (11) can be expressed as

(12) $$\int_{\Gamma }Ν\xi \frac{\partial \bar{p}}{\partial \theta }d\bar{y}-\vartheta Ν\zeta \frac{\partial \bar{p}}{\partial \bar{y}}d\theta -\iint_{\Omega }\left(\xi \frac{\partial Ν}{\partial \theta }\frac{\partial \bar{p}}{\partial \theta }+\vartheta \zeta \frac{\partial Ν}{\partial \bar{y}}\frac{\partial \bar{p}}{\partial \bar{y}}+Ν\psi \right)d\theta d\bar{y}=0$$

where Γ is the boundary of the integral domain. To tackle with the pressure distribution over the whole lubrication zone, the piston surface is discretized with triangular elements as shown in Figure 2a. In this study, the domain is discretized by employing triangular elements. Due to the mesh-dependence problem in calculating oil film pressure by FEM, adopting the appropriate mesh density is very important for both the efficiency and precision of the analysis. On the one hand, high mesh density will result in a high computational load. On the other hand, low mesh density may introduce computational accuracy and convergence problems.

The shape function of the triangular element can be expressed as

(13) $$Ν={\left[{Ν}_{1},{Ν}_{2},{Ν}_{3}\right]}^{T}$$

where

(14) $$\begin{array}{l} {Ν}_{i}=a_{i}+b_{i}\theta +c_{i}\bar{y},\;\;\;\;\\ a_{i}=\frac{1}{2\Delta }\left(\theta_{j}{\bar{y}}_{k}-\theta_{k}{\bar{y}}_{j}\right),b_{i}=\frac{1}{2\Delta }\left({\bar{y}}_{j}-{\bar{y}}_{k}\right),c_{i}=\frac{1}{2\Delta }\left(\theta_{k}-\theta_{j}\right),\\ \Delta =\frac{\left(\theta_{j}-\theta_{i}\right)\left({\bar{y}}_{k}-{\bar{y}}_{i}\right)-\left(\theta_{k}-\theta_{i}\right)\left({\bar{y}}_{j}-{\bar{y}}_{i}\right)}{2},\\ i=1,2,3;\;\;\;j=2,3,1;\;\;k=3,1,2 \end{array}$$

For the inner element $\Omega_{i}^{e}$, only the second item in Equation (12) should be considered

(15) $$\left\{\begin{array}{l} -\iint_{\Omega_{i}^{e}}\left(\xi \frac{\partial Ν}{\partial \theta }\frac{\partial \bar{p}}{\partial \theta }+\vartheta \zeta \frac{\partial Ν}{\partial \bar{y}}\frac{\partial \bar{p}}{\partial \bar{y}}\right)d\theta d\bar{y}=K_{i}^{e}\cdot {\bar{p}}_{i}^{e}\\ \iint_{\Omega_{i}^{e}}Ν\psi d\bar{x}d\bar{y}=F_{i}^{e} \end{array}\right.$$

where ${\bar{p}}_{i}^{e}={\left[{\bar{p}}_{1}^{i},{\bar{p}}_{2}^{i},{\bar{p}}_{3}^{i}\right]}^{T}$ is the dimensionless nodal pressure vector of the element i. $K_{i}^{e}$ is the element stiffness matrix, and $F_{i}^{e}$ the force vector. Both are calculated through numerical integration based on the nodal information and nodal oil film thickness.

For the boundary elements, it can be easily proved that the integration on the second boundary condition is equal to zero, while the first boundary condition can be considered by setting the known pressure on the corresponding nodes.

It should be mentioned that in this study, the piston surfaces are discretized all over with triangular elements in advance, and the piston-cylinder interface involves a changing domain and moving boundaries. Therefore, it is difficult to ensure there are always discrete nodes located on the lubrication boundaries during the relative sliding between the piston and cylinder. As shown in Figure 2a, in the initial mesh there are no nodes located on the lubrication boundaries OLE and ILE at the current moment. In order to calculate the lubrification performance accurately, a fine adjustment is made to obtain the final mesh by moving the nodes on ${\bar{y}}_{{}_{Mesh1}}$ and ${\bar{y}}_{{}_{Mesh2}}$ (the nearest nodes to the lubrication boundaries) to the lubrication boundaries, and to obtain the final mesh (shown in Figure 2b). The advantage of this method is that only the elements with the adjusted nodes should recalculate the element stiffness matrix $K_{i}^{e}$ and force vector $F_{i}^{e}$. This can improve the computational efficiency of the model.

Based on the above statement, the boundary condition stated in Equation (8) can be modified as

(16) $$\left\{\begin{array}{l} \left.\begin{array}{c} \begin{array}{l} \bar{p}(\theta ,\bar{y})=0\;\;\;\;\;\;\;\;\;\;\;\bar{y}={\bar{y}}_{o}∼\;{\bar{y}}_{\mathrm{OLE}}\\ \bar{p}(\theta ,\bar{y})={\bar{P}}_{s}\;\;\;\;\;\;\;\;\;\bar{y}={\bar{y}}_{ILE}\sim {\bar{y}}_{i} \end{array}\\ \bar{p}(0,\bar{y})=\bar{p}(2\pi ,\bar{y})\;\;\;\;\;\;\;\;\;\;\;\;\;\;\;\;\;\;\;\; \end{array}\right\}\;\;The\;1st\;boundary\;condition\\ \left.\begin{array}{l} \frac{\partial \bar{p}}{\partial \theta }(0,\bar{y})=\frac{\partial \bar{p}}{\partial \theta }(2\pi ,\bar{y})\;\;\\ \frac{\partial \bar{p}}{\partial \bar{y}}(\theta ,\bar{y})=0\;\;\;\;\;\;\;\;\;\;\bar{y}={\bar{y}}_{\mathrm{ILE}},{\bar{y}}_{OLE}\; \end{array}\right\}\;\;The\;2nd\;boundary\;condition \end{array}\right.$$

where ${\bar{y}}_{i}$ and ${\bar{y}}_{o}$ correspond to the inner and outer edge of the piston in the local coordinate system $O^{\prime }\text{-}X^{\prime }Y^{\prime }Z^{\prime }$.

By assembling the corresponding items of the elements in Equation (15), the average Reynolds equation can be transformed into Equation (17). The hydrodynamic oil film pressure $p_{h}$ can be obtained by solving this equation with the consideration of the boundary conditions.

(17) $$K\bar{p}=F$$

### 2.3. Forces and Moments Acting on the Piston

The surface is not perfectly smooth but rough and covered with asperities. The oil film thickness of the piston-cylinder interface is of the same order of magnitude as the roughness of the surfaces. When the lubricating oil film between the piston and the cylinder liner is very thin, the 2 lubricating surfaces will come into asperity contact. This has a certain impact on the lubrication performance of the piston-cylinder liner system. Therefore, apart from the hydrodynamic oil film force, the equivalent asperity contact force in mixed lubrication should also be considered. There are 2 methods for predicting the contact pressure of asperities, one being the deterministic method in which the actual measured surface profile data is used as input to characterize the rough surface [30], and the other being the statistical method which uses a series of statistics parameters to characterize rough surfaces. Due to the high computational cost of the former method, the latter method represented by the Greenwood-Tripp asperity contact model based on statistical methods is widely used in engineering. By assuming Gaussian distribution for the surface roughness, the asperity contact pressure $p_{c}$ can be calculated by using the Greenwood and Tripp asperity contact model [31,32], expressed as

(18) $$p_{c}(h)=KE^{\prime }F_{5/2}\left(\frac{h}{\sigma }\right),\;\;\;\;\;\;\;K=\left(\frac{16\sqrt{2}}{15}\right)\pi {\left(\eta_{s}\beta \delta \right)}^{2}\sqrt{\frac{\delta }{\beta }}$$

where $E^{\prime }$ is the composite elastic modulus, $\eta_{s}$ is the number of asperities per unit contact area, and β is the asperity radius of curvature. In this study, an assumed value $K=1.198\times 10^{-4}$ will be used [32,33]. *F*_5/2_(*H*) relates to the probability distribution of asperity height. For the surface roughness with Gaussian distributed asperities, *F*_5/2_ has the form of [31]

(19) $$F_{5/2}\left(H\right)=\left\{\begin{array}{ll} 4.486\times 10^{-5}{\left(4-H\right)}^{6.804} & H<4\\ 0 & H\geq 4 \end{array}\right.$$

**Figure 3:**
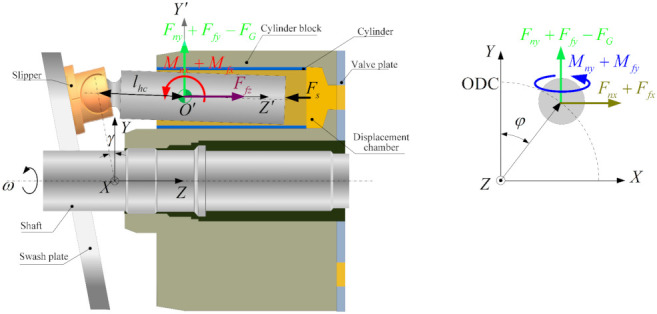
Typical structure of an axial piston pump, and forces and moment acting on the piston.

Figure 3 presents the forces and moments acting on the piston. The total normal force in the piston-cylinder interface consists of the hydrodynamic force and the asperity contact force. They are obtained by integrating the pressure on the piston surface along X-axis and Y-axis, and expressed as

(20) $$\begin{array}{l} F_{nx}=-\iint_{\Omega }\left(p_{h}+p_{c}\right)\sin\theta dxdy\\ F_{ny}=-\iint_{\Omega }\left(p_{h}+p_{c}\right)\cos\theta dxdy \end{array}$$

The total moments, from the normal pressure and acting on the head of the piston along X-axis and Y-axis, can be expressed as

(21) $$\begin{array}{l} M_{nx}=\iint_{\Omega }y\left(p_{h}+p_{c}\right)\cos\theta dxdy\\ M_{ny}=-\iint_{\Omega }y\left(p_{h}+p_{c}\right)\sin\theta dxdy \end{array}$$

The friction forces acting on the piston result from the lubricant shear stress of the oil film and asperity contacts in mixed lubrication. The asperity contact friction can be calculated based on Coulomb’s law [2]. The hydrodynamic shear stress, in both the axial and circumferential directions, can be calculated from Reynolds theory and is expressed as [21,25]

(22) $$\begin{array}{l} \tau_{z}=-\frac{\mu v}{h}\left(\Phi_{f}+\Phi_{fs}\right)-\Phi_{fp}\frac{h}{2}\frac{\partial p}{\partial y}\\ \tau_{c}=\frac{\mu u}{h}\left(\Phi_{f}+\Phi_{fs}\right)-\Phi_{fp}\frac{h}{2}\frac{\partial p}{\partial x} \end{array}$$

where $\Phi_{f}$, $\Phi_{fs}$, and $\Phi_{fp}$ are shear stress factors. Their calculation can be found in Patir and Cheng’s works [21,25]. In the above equations, the first items on the right-hand side denote the Couette contribution caused by wall movement. The second items represent the Poiseuille contribution resulting from pressure difference. Hydrodynamic friction is produced by the combined action of these 2 factors.

Therefore, the total friction force acting on the piston can be expressed as

(23) $$\begin{array}{l} F_{fx}=\iint_{\Omega }\left(\tau_{c}-\mu_{f}p_{c}\right)\cos\theta dxdy\\ F_{fy}=-\iint_{\Omega }\left(\tau_{c}-\mu_{f}p_{c}\right)\sin\theta dxdy\\ F_{fz}=\iint_{\Omega }\left(\tau_{z}-\mathrm{sign}(v)\mu_{f}p_{c}\right)dxdy \end{array}$$

and their moments acting on the head of the piston along the X-axis and Y-axis can be expressed as

(24) $$\begin{array}{l} M_{fx}=\iint_{\Omega }y\left(\tau_{c}-\mu_{f}p_{c}\right)\sin\theta dxdy\\ M_{fy}=\iint_{\Omega }y\left(\tau_{c}-\mu_{f}p_{c}\right)\cos\theta dxdy \end{array}$$

where $\mu_{f}$ is the friction coefficient of the asperity contact. This can be measured experimentally or determined by statistical elastoplastic asperity contact models, as conducted in [34]. In this work, it is assumed to be a constant of 0.13, as seen in [2,17,18,20,28].

## 3. Multibody Dynamics Model of a Swash-Plate Axial Piston Pump

### 3.1. Multibody Dynamics Model of the Pump Based on the Lagrangian Formalism

The swash-plate axial piston pump, which consists of a swash plate, slipper, cylinder, shaft, piston, and valve plate, can be regarded as a multibody dynamics system (shown in Figure 3). Based on the Lagrangian formalism [35], this section first describes the multibody dynamics model of the system to govern its behavior.

In this study, all the components of the system are assumed to be rigid, and all joints ideal (i.e., without clearance and friction) except for the piston-cylinder pair. All components are interconnected by kinematic constraints at ideal joints, while the piston-cylinder interface is handled with lubricated clearance. The existence of the clearance produces hydrodynamic force to connect the piston and the cylinder, rather than the kinematic constraint to the piston.

Based on the above assumptions, the pump is degraded into a system with 2 moving components. One is the piston with its head sliding on the swash plate. As stated in Section 2.1, the piston does not rotate along the Z-axis, and thus it can be regarded as a body with 5 degrees of freedom in the global coordinate system. The other is the cylinder which rotates along the shaft with a constant rotational speed. Therefore, the system has 6 degrees of freedom, and the generalized Cartesian coordinate can be written as

(25) $$q={\left[X_{O^{\prime }},\;Y_{O^{\prime }},\;Z_{O^{\prime }},\;\theta_{x},\;\theta_{y},\varphi \right]}^{T}\;$$

where φ is the angular displacement of the cylinder, as shown in Figure 1.

Kinematic constraint equations always exist because of mechanical joints or specified motion trajectories [35]. In this case, the kinematic constraints Φ can be written in a compact form as

(26) $$\Phi \left(q,t\right)=\left(\begin{array}{l} (Y_{O^{\prime }}+l_{hc}\theta_{x})\tan\gamma +l_{hc}-Z_{O^{\prime }}\\ \varphi -\omega t \end{array}\right)=0$$

Differentiating Equation (26) twice to time yields the acceleration constraint equation

(27) $$\ddot{\Phi }={\left(\Phi_{q}\dot{q}\right)}_{q}\dot{q}+\Phi_{q}\ddot{q}+2\Phi_{qt}\dot{q}+\Phi_{tt}=0$$

where $\Phi_{q}$ is the Jacobian matrix of the constraint equations. $\dot{q}$ and $\ddot{q}$ are vectors of the system velocity and acceleration, respectively.

The dynamics equations for a constrained multibody system of rigid bodies can be written as [35,36]

(28) $$M\ddot{q}=Q-\Phi_{q}^{T}\lambda$$

where Q is the generalized force vector and can be expressed as

(29) $$Q={\left[F_{nx}+F_{fx},F_{ny}+F_{fy}-F_{G},F_{fz}-F_{s},M_{nx}+M_{fx},M_{ny}+M_{fy},M_{shaft}\right]}^{T}$$

where $F_{G}$ is the gravity of the piston, and $F_{s}$ the supply pressure force. The others are the hydrodynamics forces and moments in the piston-cylinder tribo-pair, as stated in Section 2.3. Due to the kinematic constraint exerted on the cylinder block (stated in Equation (26)), the values of the moment acting on the shaft ($M_{shaft}$), as well as the inertia moment of the cylinder about Z-axis ($J_{\mathrm{cz}}$), will not affect the solution of the system. The vector λ is the Lagrange multiplier, and the term $-\Phi_{q}^{T}\lambda$ can be used to evaluate the joint reaction forces [37].

The mass matrix of the system can be expressed as

(30) $$M=\mathrm{diag}(m_{p},\;m_{p},\;m_{p},\;J_{px},\;J_{py},\;J_{\mathrm{cz}})$$

where $m_{p}$ is the mass of the piston. $J_{px}\;$ and $\;J_{py}$ are the inertia moments of the piston corresponding to its COM about the X-axis and Y-axis, respectively. $J_{\mathrm{cz}}$ is the total inertia moments of the cylinder about Z-axis.

Combining Equations (27) and (28), the dynamics equations of a system subject to constraints can be stated in the form [14,35,36,37,38,39]

(31) $$\left[\begin{array}{cc} M & \Phi_{q}^{T}\\ \Phi_{q}^{} & 0 \end{array}\right]\left[\begin{array}{c} \ddot{q}\\ \lambda \end{array}\right]=\left[\begin{array}{c} Q\\ \gamma \end{array}\right]$$

where $\gamma =\Phi_{q}\ddot{q}=-{\left(\Phi_{q}\dot{q}\right)}_{q}\dot{q}-2\Phi_{qt}\dot{q}-\Phi_{tt}$ can be obtained from Equation (27).

The equilibrium equations, represented by Equation (31), do not make explicit use of the position and velocity equations associated with the kinematic constraints. Therefore, due to numerical errors there it is not assured that the system constraints are fulfilled during the forward dynamic integration of the system velocities and accelerations [14,39,40,41]. To control the constraints violation during numerical integration, this study adopts the Baumgarte stabilization technique which damps out the acceleration constraint violations by feeding back the violations of positions and velocity constraints [14,15,39,40,41]. Equation (31) can be modified as

(32) $$\left[\begin{array}{cc} M & \Phi_{q}^{T}\\ \Phi_{q} & 0 \end{array}\right]\left[\begin{array}{c} \ddot{q}\\ \lambda \end{array}\right]=\left[\begin{array}{c} Q\\ \gamma -2\chi \left(\Phi_{q}\dot{q}+\Phi_{t}\right)-\kappa^{2}\Phi \end{array}\right]$$

where χ and κ are termed as feedback parameters which should be arbitrarily chosen [15,42].

### 3.2. Computational Algorithm

**Figure 4:**
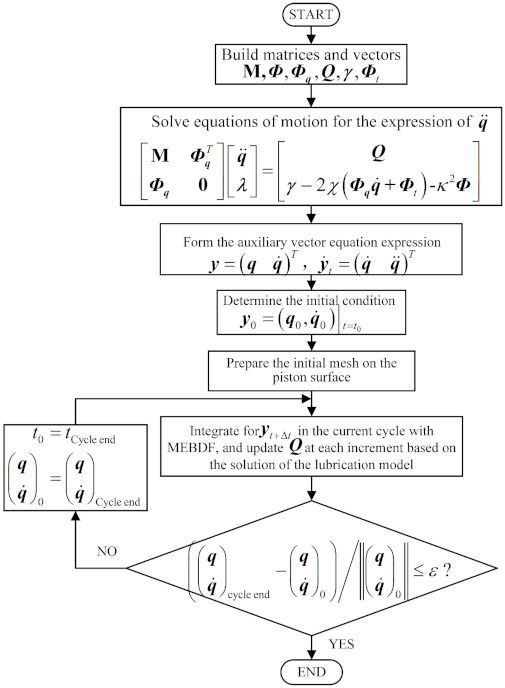
Flowchart of the computational procedure for the analysis of the system.

The computational scheme for the analysis of the pump system is shown in Figure 4, and is described as follows:

**Step 1:** According to the geometry and physical properties, the constraint conditions of the pump system, and the generalized external force, the dynamics equations of the system can be established as shown in Equation (32). The equations are a set of n second-order ordinary differential equations (ODEs). Then, the expression of $\ddot{q}$ can be derived as function of q, $\dot{q}$, Q, and t. In this step, q, $\dot{q}$, Q, and t are all symbols used to express $\ddot{q}$.

**Step 2:** By introducing 2 new vectors $y={\left(q\;\;\;\dot{q}\right)}^{T}$ and $\dot{y}={\left(\begin{array}{cc} \dot{q} & \ddot{q} \end{array}\right)}^{T}$, the n second-order differential equations of motion can be converted into 2n first-order ODEs. The solving of motion equations for the pump systems can be regarded as an initial value problem with the form of $\dot{y}=f\left(y,t\right)$, $y(0)=y_{0}$.

**Step 3:** The whole surface of the piston with triangular elements is discretized, and the initial mesh is obtained. Then the initial conditions of the system $y_{0}=\left(q_{0},{\dot{q}}_{0}\right)\left|{}_{t=t_{0}}\right.$ is determined, and the integration process for the current full cycle is initiated by using the modified extended backward differentiation formulae (MEBDF) [43,44]. At each integration step, according to the current motion states of the system (i.e., the motion of the piston relative to the cylinder), the oil film and asperity contact pressure can be calculated through the average Reynolds equation (Equation (1)). Subsequently, the oil film forces and moments exerted on the piston can be calculated according to Equations (20)–(24), and the external force vector Q is updated. Then, the motion state of the system at the next integration step can be determined with MEBDF. Based on the updated relative motion of the piston and cylinder, the integration process is repeated until the current full cycle is completed.

**Step 4:** Repeat **Step 3** until the deviation between the initial values and the final values of the current cycle are within the allowable range.

Apart from the load-carrying ability and friction power loss from the piston-cylinder interface, the leakage of the gap flow is also revealing. The flow from the displacement chamber through the gap into the case of the pump results in volumetric losses and must be kept as small as possible. After the pressure distribution on oil film is obtained, the leakage flow can be derived from the integration of the film velocity distribution in the axial direction and expressed as [45]

(33) $$Q_{Leakage}=\int_{0}^{2\pi R_{p}}\int_{0}^{h}v\frac{z}{h}+\frac{1}{2\mu }\frac{\partial p}{\partial y}\left(z^{2}-hz\right)dzdx$$

The former term denotes the Couette contribution, and the latter represents the Poiseuille effect. Due to the continuity of the Reynolds equation, the flow is constant over the entire fluid film length for each cross-section.

## 4. Model Validation

**Figure 5:**
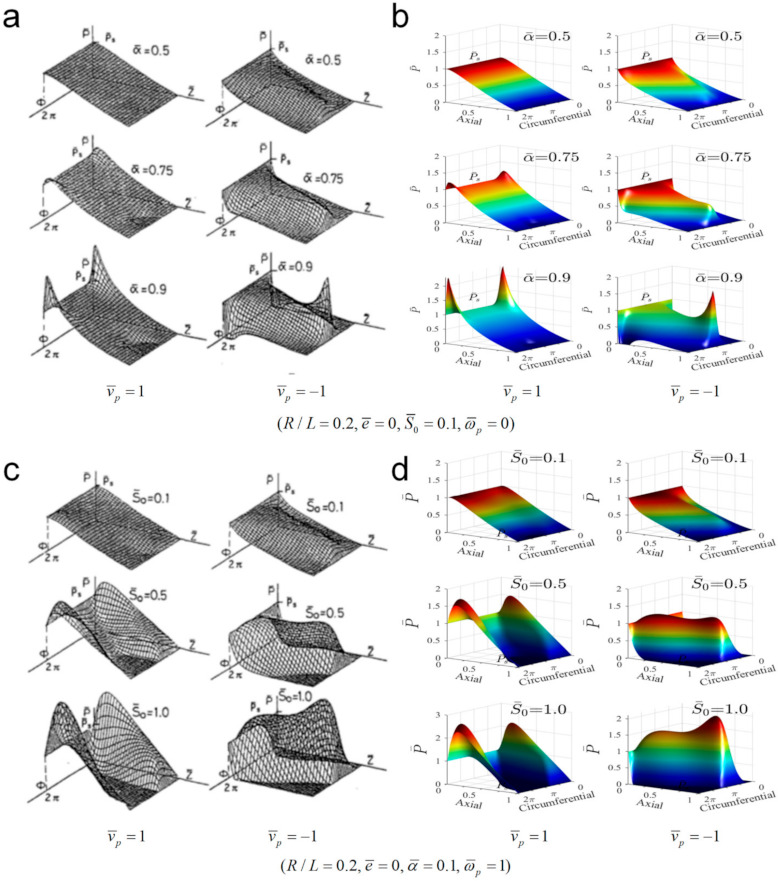
Distribution of oil film pressure between the piston and cylinder: (**a**) Results from [3] without considering piston spinning, (**b**) the current results without considering piston spinning, (**c**) results from [3] considering piston spinning, and (**d**) the current results considering piston spinning.

By neglecting the transient effect, the steady-state analysis capacity of the current transient lubrication model is first validated with a steady-state model as reported in [3] (as shown in Figure 5). In the figure, parameters R and L are the radius and lubricated length of the piston, respectively. $\bar{e}=e/C$ denotes the non-dimensional eccentricity of the piston, and $\bar{\alpha }=\alpha /\left(2C/L\right)$ the non-dimensional misalignment angle of the piston. ${\bar{v}}_{p}$ and ${\bar{\omega }}_{p}$ are the non-dimensional axial and spinning angular velocities of the piston, respectively. The parameter ${\bar{S}}_{0}=\left(\mu vR/C^{2}\right)/P_{s}$ represents the ratio of the dynamic pressure due to the wedge effect to the supply pressure $P_{s}$ in the cylinder.

Figure 5a,c shows the oil film pressure distribution calculated in [3]. The former shows the oil film pressure distribution under different piston misalignments without considering piston spinning. When the piston moves in the inward stroke with the dimensionless axial velocity of 1, a peak of pressure near the piston chamber increases with the increase of the eccentricity. Likewise, in the piston outward stroke with the same velocity, a pressure peak near the front end of the piston increases with the increase of the eccentricity. Figure 5c shows the effect of ${\bar{S}}_{0}$ on oil film pressure with the consideration of piston spinning. When ${\bar{S}}_{0}$ is small, the wedge effect as a result of the piston motion is insignificant and the oil film pressure is dominated by the supply pressure $P_{s}$.

While with the increase of ${\bar{S}}_{0}$, the dynamic pressure becomes prominent and the region with high pressure expands both in pumping and suction strokes. Figure 5b,d presents the oil film pressure distributions derived from the current model, without or with piston spinning, respectively. On comparing Figure 5a,b as well as Figure 5c,d, the satisfactory result validates the steady-state analysis ability of the current model.

**Figure 6:**
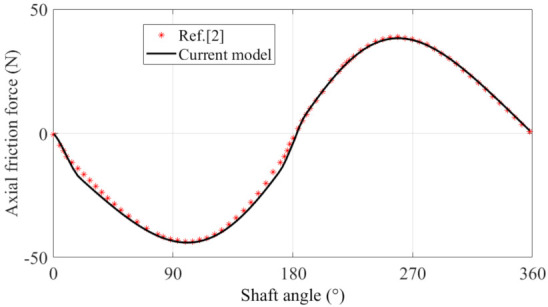
Transient axial friction force in comparison with the result from [2].

The proposed model is further validated by comparing the axial friction force with a transient model reported in [2] (as shown in Figure 6). Here, the operating conditions are a 16 MPa pump outlet pressure and 1500 rpm shaft speed. The clearance between the piston and cylinder is 0.01 mm, and more detailed parameters can be found in [2]. As stated in Equation (22), the hydrodynamic friction is produced by the combined action of Couette and Poiseuille flow. The former is mainly related to the axial velocity of the piston and dominates in the axial hydrodynamics friction force, as presented in the figure. The latter concerns mainly the pressure difference between the 2 ends of the piston. Since the supply pressure in the pumping stroke is higher than that of the suction stroke, the Poiseuille stress in the pumping stroke brings about more axial hydrodynamic friction force than in the suction stroke, and thus causes higher axial friction force. This phenomenon has also been found in [2]. The axial hydrodynamic friction predicted from the current transient shows good agreement with that reported in [2], which also validates the feasibility of the proposed approach in predicting piston-cylinder lubrication performance in a swash-plate axial piston pump.

## 5. Results and Discussion

The proposed transient model was performed on a swash−plate axial piston pump with a variable−length piston-cylinder interface. Investigations were performed into the lubrication and leakage performances of the piston-cylinder interface, as well as their dependence on cylinder length and the tilt angle of the swash plate.

### 5.1. The Performance of the Piston-Cylinder Interface with Variable Lengths

**Figure 7:**
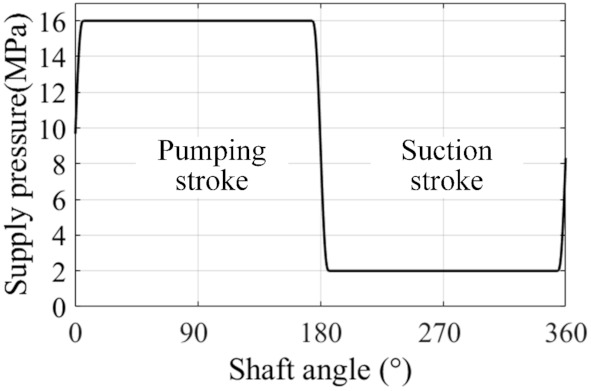
The supply pressure inside the cylinder chamber.

Some necessary input parameters for the simulation are listed in Table 1, and the instantaneous supply pressure from the displacement chamber is illustrated in Figure 7. This determines the boundary conditions for the pressure distribution and the external force exerted on the piston. The simulation parameters for this system are shown in Table 2. $N_{y}$ and $N_{x}$ are the number of elements along the axial and circumferential directions in the domain Ω (shown in Figure 2), respectively. Compared with the case meshed with four-fold elements (i.e., $2N_{x}^{}\;\times \;2N_{y}^{}$), this mesh density can improve the solving efficiency for hydrodynamic lubrication by about 50%, with the results remaining about the same. Therefore, it can be proved that this mesh is convergent and can guarantee good accuracy.

**Figure 8:**
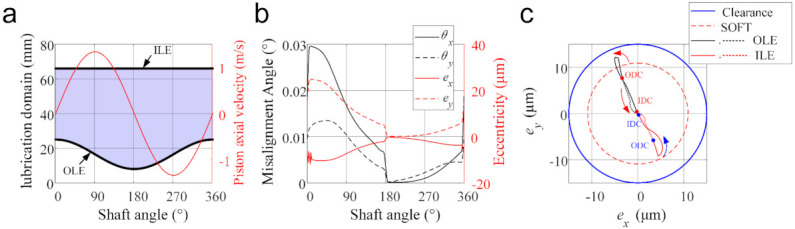
Dynamics results of the piston-cylinder pair: (**a**) Lubrication domain and the piston axial velocity, (**b**) Piston-oscillating eccentricities micro-motion, and (**c**) Center trajectories of OLE and ILE relative to the cylinder center.

Subsequently, the numerical results regarding the dynamics, tribological and internal leakage performances in the piston-cylinder interface are presented and analyzed. Figure 8 presents the dynamics behaviors of the mechanism. In the current case, the piston-cylinder interface length varies with the piston motion, as shown in Figure 8a. The outer lubrication edge (OLE) moves along the piston, while the inner lubrication edge (ILE) remains unchanged because the cylinder is long enough to ensure this feature. The outer dead center (ODC) is the position where the piston is farthest away from the valve plate at a 0° shaft angle (SA), and the piston-cylinder interface has the smallest lubrication domain. At the inner dead center (IDC), the rotation angle of the cylinder is 180° SA, and the piston-cylinder interface has the largest lubrication domain. The piston takes a periodic reciprocating-rotating motion in the axial direction, and its axial velocity performs a sinusoidal form reaching zero at dead centers (ODC and IDC).

**Figure 9:**
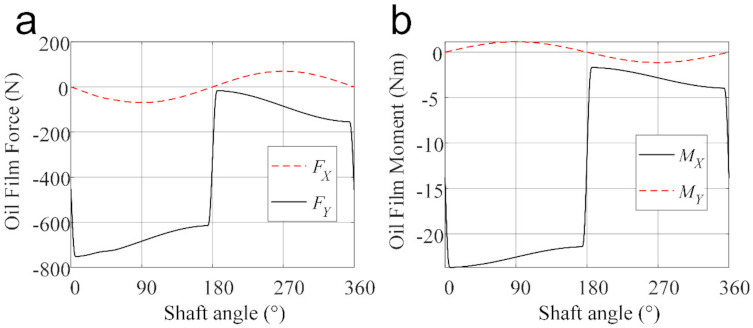
The oil film forces and moment acting on the piston: (**a**) The resultant oil film forces, and (**b**) the resultant oil film moments acting on the COM of the piston.

Figure 8b depicts the dynamic oscillatory behavior of the piston-cylinder interface, i.e., the eccentric micro-motion of the COM of the piston, as well as the misalignment angle of the piston over one shaft revolution. The piston self-adjusts the eccentricities and misalignments to balance the external oscillating load, which derives mainly from the displacement chamber supply pressure. In the pumping stroke, it is visible that the eccentricities and misalignments are larger than those in the suction stroke and drop precipitously at 180° SA. The reason is that the higher chamber pressure in the pumping stroke produces higher vertical reaction force and higher moment acting on the piston (shown in Figure 9), which requires smaller oil film thickness and thus larger oil film pressure to separate the piston from the cylinder. The similar results were also obtained by [2,5].

Figure 8c shows the center trajectories of OLE and ILE on the piston relative to the cylinder center. It can be found that the centers of OLE and ILE mainly concentrate on the second and fourth quadrants in the figure, respectively. This can easily be concluded from the micro-motion of the piston shown in Figure 8b, where the misalignment angles $\theta_{x}$ and $\theta_{y}$ are positive. Moreover, in the current case, there are micro-asperity contacts between the piston and cylinder on the OLE during a period, because the distance between the centers of OLE and cylinder is smaller than the soft oil film thickness (SOFT).

Figure 9 shows the variations of total oil film forces acting on the piston and moments to the COM of the piston. It is clear that in the pumping stroke, the component of oil film force in Y-axis direction ($F_{Y}$) is much larger than that in X-axis direction ($F_{X}$), and the fluid moment to the COM of the piston is much higher relative to the X-axis than that relative to the Y-axis. This is because the reaction force from the swash plate in the Z-axis direction functions as the main force in balancing the chamber pressure, and that the reaction force from the swash plate in the Y-axis direction is the main factor for the misalignment and inclination of the piston. Thus, $F_{Y}$ and $M_{X}$ are larger than $F_{X}$ and $M_{Y}$ for balancing the piston, respectively. Besides, the centrifugal force of the piston also contributes to the resultant oil film forces and moments, which causes $F_{X}$ and $M_{Y}$ to perform as sine curves. This phenomenon has also been observed in [2].

**Figure 10:**
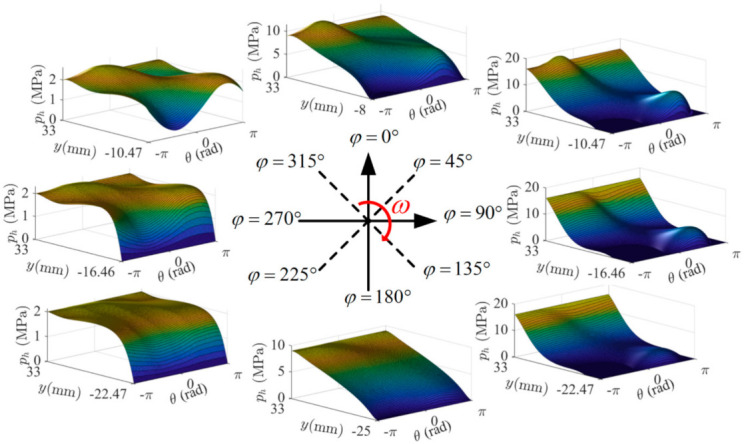
The oil film pressure distribution over 1 shaft revolution.

Figure 10 demonstrates the variation of hydrodynamic pressure distribution in the piston-cylinder interface over one shaft revolution. Due to the misalignment, as well as the squeezing effect caused by the radial micro-motion of the piston relative to the cylinder (shown in Figure 8b), there are two pressure peaks at ODC (i.e., the moment of 0° SA). Then, the supply pressure suddenly increases to the maximum, and both peaks increase, as shown by the moment of 45° SA. The further increase of the shaft angle increases the lubrication area, and thus produces a reduction in peak pressures at 90° SA. At IDC (i.e., the moment of 180° SA), the supply pressure is decreased, and the lubrication area is the largest, which makes the pressure distribution gentle. As the piston moves towards the ODC, the lubrication area decreases gradually, and the increase of the misalignment angle of the piston (as shown in Figure 8b) leads the pressure peaks to appear again.

**Figure 11:**
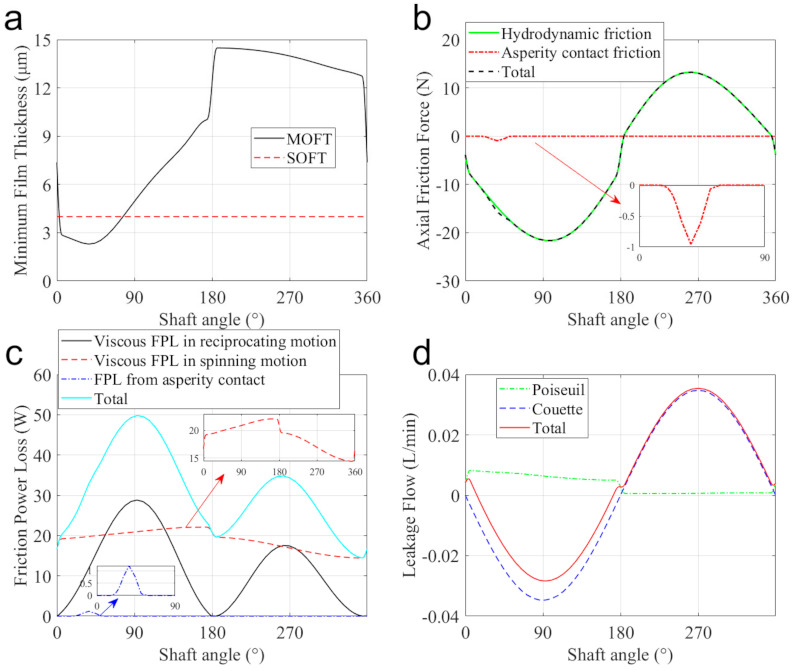
Tribological performances of the piston/ cylinder interface over 1 revolution: (**a**) minimum oil film thickness, (**b**) axial friction force, (**c**) friction power loss, and (**d**) leakage flow.

Figure 11a presents the minimum oil film thickness (MOFT) in the clearance of the piston-cylinder tribo-pair over one revolution. It can be found that from 0° to about 10° SA, the MOFT decreases significantly. This is because the sharp increase in the supply pressure and the small sliding velocity at this stage require a small fluid thickness and thus high hydrodynamic pressure to balance the increasing external force and moment (shown in Figure 9). Afterward, the MOFT still decreases slightly and reaches the minimum at about 37° SA due to the increase of the inclination of the piston (shown in Figure 8b). From 37° to 175° SA, the MOFT increases continuously with the increase in the lubrication area (shown in Figure 8a). From 175° to 185° SA, the MOFT rises steeply because of the sharp decrease in the supply pressure and reaches its maximum at 185° SA. After that, in the suction stroke, the MOFT decreases along with the lubrication area.

**Figure 12:**
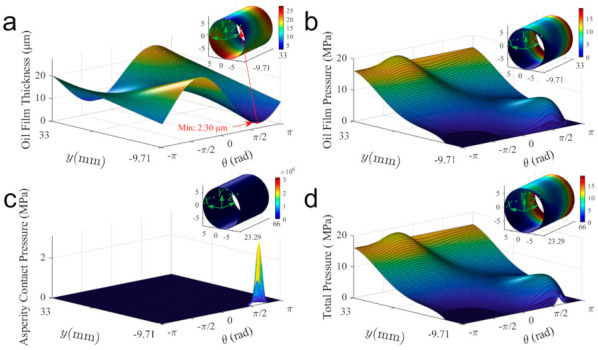
The oil film thickness and pressure distribution at about 37° SA: (**a**) the oil film thickness, (**b**) the oil film pressure, (**c**) the micro-asperity contact pressure, and (**d**) the total contact pressure.

Besides, as can be seen from the figure, the piston-cylinder interface is fully lubricated most of the time. Although MOFT reaches its minimum value at around 37° SA, there is no direct contact in the tribo-pair due to the asperity contact on surfaces that prevents the piston from further approaching the inner surface of the cylinder. Figure 12 shows the fluid film thickness and pressure distribution at 37° SA to describe the mixed lubrication state at this moment. It can be found that due to the misalignment and inclination of the piston, the MOFT occurs on the OLE (the inset in Figure 12a presents this position clearly). The total pressure consists of hydrodynamic pressure and micro-asperity contact pressure. The hydrodynamic pressure presents two pressure peaks, as stated in Figure 10. The asperity contact pressure (shown in Figure 12c) concentrates on the position where the MOFT reaches its minimum and contributes to the friction force and frictional power loss (FPL), as shown in Figure 11b,c.

Compared with the suction stroke, the higher supply pressure in pumping stroke produces a thinner oil film (shown in Figure 11a), and thus higher shear stress (stated in Equation (22)). This causes higher axial friction force and viscous FPL, as shown in Figure 11b,c.

The leakage across the piston-cylinder clearance is shown in Figure 11d. The negative value denotes a flow from the case into the displacement chamber and the positive value from the chamber into the case. In the current case, the Couette flow dominates and shows a positive correlation with the axial velocity of the piston. The Poiseuille flow results from the pressure difference and induces leakage flow from the displacement chamber into the case. Therefore, in the pumping stroke, the Poiseuille effect is more obvious than that in the suction stroke.

### 5.2. The Influence of the Cylinder Length

**Figure 13:**
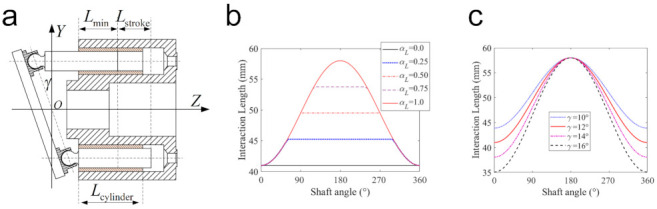
(**a**) The schematic of a swash-plate pump with different cylinder lengths and tilt angles, (**b**) the length of the lubrication interface with different cylinder lengths, and (**c**) the length of the lubrication interface with different tilt angles of the swash plate.

The cylinder length determines the effective lubrication region and has a significant impact on the performance of the piston-cylinder interface. As shown in Figure 13a, if the cylinder length $L_{cylinder}$ is not larger than $L_{min}$, the pump runs with a constant piston-cylinder interface length (denominated as pump interface C [9]). Instead, the piston-cylinder interface length is no longer a constant and varies with the piston motion. In this subsection, a ratio of $\alpha_{L}=\left(L_{cylinder}-L_{min}\right)/L_{stroke}$ is introduced, and five cases with different $\alpha_{L}$ values of 0.0, 0.25, 0.50, 0.75, and 1.0 are simulated to reveal the impact of the cylinder length. Other structure parameters remain the same as those listed in Table 1. The results for the interface length between the piston and the cylinder are shown in Figure 13b, in which it is clear that with the increase in the cylinder length, the pump evolves from pump interface C into pump interface V (i.e., the pump with a varying piston-cylinder interface length all over the stroke [9]).

**Figure 14:**
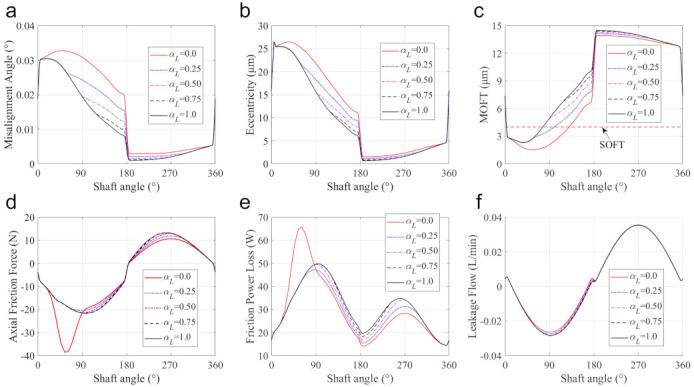
Performances of the pump with different cylinder lengths: (**a**) the misalignment angle, (**b**) the eccentricity, (**c**) the MOFT, (**d**) the axial friction force, (**e**) the friction power loss, and (**f**) the leakage flow.

**Figure 15:**
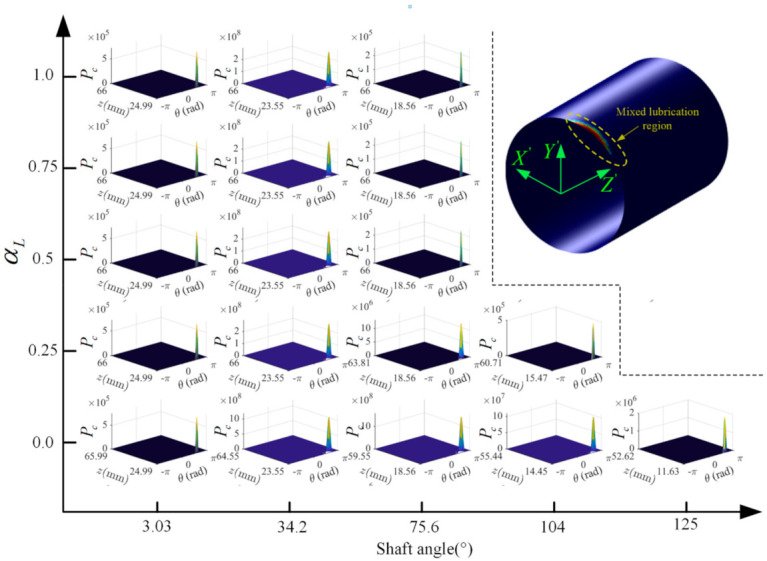
Variation of the mixed lubrication region with the shaft angle in cases with different cylinder lengths.

The effects of cylinder length on dynamics, lubrication, and leakage performances, are shown in Figure 14. Under the same supply pressure, the increase in cylinder length can enlarge the lubrication interface. This can improve the stability of the piston by reducing the misalignment and eccentricity of the piston, especially in the pumping stroke (shown in Figure 14a,b). Furthermore, this stability improvement of the piston is also conducive to raising the MOFT and reducing the duration of the mixed lubrication, as shown in Figure 14c. It can be found that in comparison with other cases, the pump with interface C ($\alpha_{L}=0$) produces the smallest MOFT, accompanied by the largest mixed lubrication duration from 3.03 to 125° SA. Figure 15 presents the variation of the asperity contact in mixed lubrication with the shaft angle in detail. The length of the lubrication region in the case of interface C remains unchanged, and due to the misalignment and inclination of the piston, the mixed lubrication mainly occurs on the OLE. For other cases, they also start to fall into mixed lubrication at about 3.03° SA, with almost the same mixed lubrication until about 62.0° SA. This is because with the increase of the shaft angle, the piston moves towards the displacement chamber, and the piston end of $\alpha_{L}=0.25$ first leaves the lubrication area with the cylinder at about 62.0° SA due to its shorter cylinder length. After that, the length of the lubrication region remains unchanged. For the rest cases ($\alpha_{L}$ equal to 0.5, 0.75, and 1.0), the mixed lubrication region only occurs in the range from 3.03 to 75.6° SA. Their cylinder can ensure the piston is always in lubrication with the cylinder in this range, thereby exhibiting the same mixed lubrication performance.

Figure 14d,e present axial friction force and total friction power loss, which is derived from the micro-asperity contact and hydrodynamic friction. Interface C produces the largest micro-asperity friction force and friction power loss. In other cases, the asperity friction force is not significant, and the hydrodynamic friction increases with the cylinder length because the hydrodynamic friction is mainly determined by sliding velocity and the interaction area. These results indicate that a longer cylinder length can improve the stability of the piston and the wear resistance because of the higher MOFT, but it can also lead to a higher friction force and a higher power loss.

**Figure 16:**
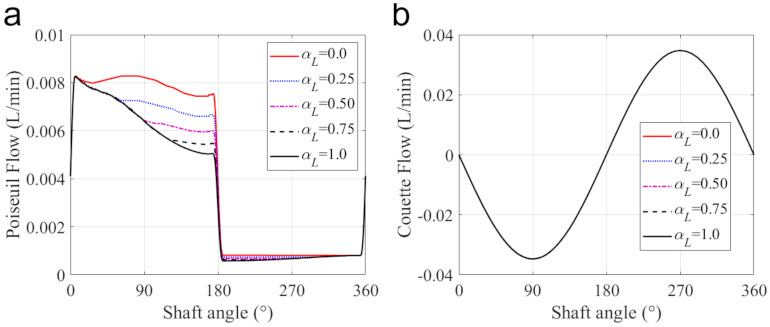
The components of leakage flow with the different cylinder lengths: (**a**) the Poiseuil flow, (**b**) the Couette flow.

The leakage flow across the piston-cylinder interface is shown in Figure 14f, and its components in the case with different cylinder lengths are shown in Figure 16. For the selected five cases, the Couette flow is almost the same because the Couette effect mainly depends on the axial velocity of the piston. However, the decrease in the cylinder length can enlarge the pressure difference of the fluent along with the piston axial, which makes the Poiseuille flow more apparent, and induces leakage flow from the displacement chamber into the case, especially in the pumping stroke.

### 5.3. The Influence of the Tilt Angle of the Swash-Plate

The tilt angle of the swash plate affects the stroke and reciprocating speed of the piston, and thus the dynamics and lubrication characteristics. In this subsection, the simulation for pump interface V with 4 tilt angles of 10 °, 12°, 14°, and 16°, are conducted. Other structure parameters remained unchanged as listed in Table 1.

**Figure 17:**
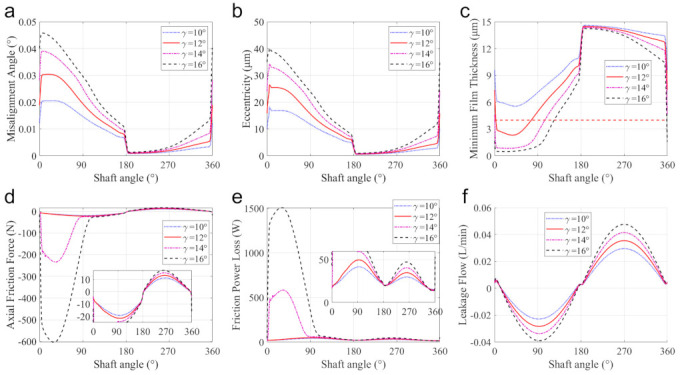
The effects of tile angles of the swash plate on: (**a**) the misalignment angle, (**b**) the eccentricity, (**c**) the MOFT, (**d**) the axial friction force, (**e**) the friction power loss, and (**f**) the leakage flow.

Figure 17 presents the effects of the tilt angle of the swash plate on the dynamics, lubrication, and leakage performances of the piston-cylinder interface. Under the same supply pressure, the increase in the tilt angle can enlarge the side force of the swash plate on the piston. This can aggravate the misalignment and the eccentricity of the piston, especially in the pumping stroke (shown in Figure 17a,b). Besides, the increase of the tilt angle can diminish the lubrication interface length (shown in Figure 13c), which can also reduce the stability of the piston and thus enlarge its inclination.

For the above-mentioned reasons, the increase of the tilt angle can reduce the MOFT (especially in the pumping stroke), which can dramatically increase asperity contact-induced friction and thus the corresponding friction power loss. According to Equation (22), the reduction of the oil film thickness can increase the hydrodynamic friction. Furthermore, the increase of the tilt angle enlarges the axial velocity of the piston, and thus the axial viscous friction force. However, the shortened lubrication interface due to the increase in the tilt angle (shown in Figure 13c) can reduce the viscous FPL in the spinning motion in some moments, especially in the suction stroke.

The increase of the tilt angle of the swash plate can shorten the lubrication interface length while enlarging the piston stroke and axial velocity. The shortened interface can make the Poiseuille effect more apparent, especially in the pumping stroke. The enlarged piston stroke and axial velocity can enhance the Couette effect. The above two effects can increase the leakage from the displacement chamber into the case, as shown in Figure 17d.

Above all, increasing the tilt angle of the swash plate proves a convenient and effective way to increase the pump displacement, but it can easily lead to serious friction, wear, and leakage problems. Therefore, in practice, it is necessary to limit the range of tilt angle of the swash plate.

## 6. Conclusions

In this study, a new approach is proposed for modeling mixed lubricated piston-cylinder pairs of variable lengths in swash-plate axial piston pumps. The approach couples the hydrodynamic mixed lubrication model of the piston-cylinder interface with the multibody dynamics model of the pump system. This approach can calculate the eccentric motion of the piston, consider the effect of roughness on lubrication performance in the mixed lubrication regime, and simulate the lubrication inside the varying piston-cylinder interface, involving a changing domain and moving boundaries. The lubrication and leakage performances of a piston-cylinder interface in a swash-plate axial piston pump, as well as their dependence on cylinder length and the tilt angle of the swash plate, are investigated. Some conclusions can be drawn as follows:

(1) Increasing the cylinder length can enlarge the lubrication interface. This can improve the stability of the piston by reducing the misalignment and eccentricity of the piston, especially in the pumping stroke.
(2) Increasing the cylinder is conducive to improving the wear-resistance of the piston by raising the minimum oil film thickness and reducing the duration of the mixed lubrication, but it can aggravate the friction force and thus the frictional power loss.
(3) In practical applications, although enlarging the tilt angle of the swash plate can effectively increase the pump displacement, it can easily lead to serious friction, wear, and leakage problems. Consequently, the tilt angle of the swash plate should be carefully selected.

In addition, it should be highlighted that the focus of this study is on presenting a new numerical method for modeling mixed lubricated piston-cylinder interfaces of variable lengths in swash-plate axial piston pumps. The elastic deformation of piston and cylinder due to oil film pressure is ignored. The thermal distortions of the piston and the cylinder, as well as the dependence of the lubricant viscosity on the pressure and temperature, are also not considered. Furthermore, the micro surface shaping [46] and texture [47] on the interface are intended to improve the lubrication performance of the piston system. This is also not considered. In our future work, elastic and thermal deformations, the dependences of the viscosity on pressure and temperature, and the surface texture design for the piston and cylinder will be further studied.

## Figures and Tables

**Table 1:** Parameters for the piston-cylinder assembly used in the analysis.

Parameters	Values
Piston radius, $R_{p}$	8 mm
Clearance, c	0.015 mm
Piston Length, $L_{p}$	66 mm
Cylinder length, $L_{cylinder}$	66 mm
Distance between the head to the COM of the piston, $L_{hc}$	33 mm
Fluid viscosity, μ	0.03 Pa·s
Shaft speed, ω	1500 r/min
The tilt angle of the swash plate, γ	12°
Piston mass, $m_{p}$	7.05 × 10^−2^ kg
Cylinder block pitch radius, $R_{\mathrm{pitch}}$	40 mm
The roughness of the piston, $\sigma_{piston}$	1.65 × 10^−7^ m
The roughness of the cylinder, $\sigma_{cylinder}$	8.15 × 10^−7^ m

**Table 2:** Simulation parameters for the system.

Baumgarte coefficients, χ, κ	1500
Minimum time step size for integration in MEBDF	1.0 × 10^−7^ s
Maximum order for integration in MEBDF	6
Number of elements in the lubrication domains, $N_{x}^{}\;\times \;N_{y}^{}$	40 × 40
Convergence criterion, ε	1%

## Data Availability

All data included in this study are available upon request by contact with the corresponding author.

## Nomenclature

c	Radial clearance
$F_{nx},\;F_{ny}$	Total normal force
$F_{fx},\;F_{fy},\;F_{fz}$	Total friction force
$F_{G}$	The gravity of the piston
$F_{s}$	Supply pressure force
h	Oil film thickness
$l_{hc}$	Distance between the head to the COM of the piston
$L_{p}$	Piston Length
$L_{cylinder}$	Cylinder length
t	The time
v, u	The axial and circumferential sliding velocity of the piston relative to the cylinder
$Q_{Leakage}$	Leakage flow
$P_{s}$	Supply pressure from the displacement chamber
$R_{c}$	The inner radius of the cylinder
$R_{P}$	The radius of the piston
$R_{Pitch}$	The pitch radius
$\left(X_{O^{\prime }},Y_{O^{\prime }},Z_{O^{\prime }}\right)$	The position of the COM of the piston in the global coordinate system
K	Stiff matrix of the Reynold equation with FEM
M	System mass matrix
$M_{nx},\;M_{ny}$	The total moments from the normal pressure
$M_{fx},\;M_{fy}$	The total moments from the shear stress
Ν	Shape function of discrete elements
p	Oil film pressure
$p_{c}$	Asperity contact pressure
Q	Generalized force vector
q	Generalized Cartesian coordinates of the system
$m_{p}$	Mass of the piston
$J_{px}\;$, $\;J_{py}$	Inertia moments of the piston corresponding to its com
x, y	Local coordinate axis along the circumferential direction and axial direction of the unwrapped fluid film thickness
ω	The shaft speed
ε	Convergence criterion
μ	Dynamic viscosity of oil
$\phi_{x}$, $\phi_{y}$	Pressure flow factors
$\tau_{z},\;\tau_{c}$	Hydrodynamic shear stress, in both axial and circumferential directions
$\phi_{s}$	Shear flow factor
$\Phi_{fs}$, $\Phi_{fp}$	Shear stress factors
$\phi_{c}$	Contact factor
$\Phi_{f}$	Term to average the sliding velocity component of the shear stress
σ	Composite roughness of the surface
$\sigma_{\mathrm{piston}}$, $\sigma_{\mathrm{cylinder}}$	The roughness of the piston and cylinder, respectively.
θ	Angular coordinate in $O^{\prime }\text{-}X^{\prime }Y^{\prime }Z^{\prime }$
$\theta_{x}$, $\theta_{y}$	The inclination angle of the piston axis in O-XYZ.
φ	Shaft angle of the pump
Ω	The lubrication domain
Γ	The boundary of an integral domain
$\mu_{f}$	The friction coefficient of the asperity contact
Φ	Kinematic constraints
λ	Lagrange multipliers
χ, κ	Feedback parameters in Baumgarte’s approach
γ	The tilt angle of the swash plate
Coordinate systems	
O-XYZ	The global coordinate system of the multibody system
$O^{\prime }\text{-}X^{\prime }Y^{\prime }Z^{\prime }$	The local coordinate system fixed on the COM of the piston and parallel to the global coordinate system
